# Antiviral Activity against Respiratory Syncytial Virus of Polysaccharide from Jerusalem Artichoke (*Helianthus tuberosus* L.)

**DOI:** 10.1155/2022/1809879

**Published:** 2022-09-20

**Authors:** Xinhuan Wan, Zihao Liu, Yuliang Wang, Yiming Yin, Xiaoqing Cai, Lina Gao, Qi Wang, Ying Li, Changzheng Zhou

**Affiliations:** ^1^School of Pharmacy, Shandong University of Traditional Chinese Medicine, Jinan 250355, China; ^2^Lunan Pharmaceutical Group Co., Ltd., State Key Laboratory of Generic Manufacture Technology of Chinese Traditional Medicine, Linyi 276006, China; ^3^Shandong Analysis and Test Center, Qilu University of Technology (Shandong Academy of Sciences), Jinan 250014, China

## Abstract

Jerusalem artichoke (*Helianthus tuberosus* L.) polysaccharide (JAP) is a chain polysaccharide composed of D-fructose connected by *β* (1-2) glycosidic bonds, which is a kind of inulin. This study evaluated the anti-respiratory syncytial virus (RSV) activity of JAP in *vivo* and in *vitro*. To investigate its antiviral activity, an MTT assay, q-PCR, enzyme-linked immunosorbent assay (ELISA), and lung histological observation were performed. The results showed that JAP showed anti-RSV activity in *vitro* with a half maximal inhibitory concentration (IC_50_) of approximately 29.15 *μ*g/mL. *In vivo* results suggested that JAP could effectively inhibit RSV proliferation in the lungs and improve lung tissue lesions in RSV-infected mice. Additionally, JAP could also reduce the expression of TLR3 and TLR4 in the lungs, increase serum anti-inflammatory factors IL-4 levels, and reduce pro-inflammatory factors TNF-*α* and TNF-*β* levels, which may be related to its anti-RSV activity. This study provides a new approach to anti-RSV therapy and enriches the potential applications of JAP.

## 1. Introduction

Respiratory syncytial virus (RSV), an important RNA virus, belongs to the genus Pneumovirus of the family Paramyxoviridae, which frequently causes lower respiratory tract infections in infants, the elderly, and adults with weakened immunity [1–4]. According to statistics, in 2015, the number of children under 5 years old who suffered from acute lower respiratory tract infection due to RSV was as high as 33.1 million, of which about 3.2 million required hospitalization, and the death toll was as high as 120,000 [2]. In addition, the annual mortality rate due to RSV infection in adults over the age of 65 in the United States is 0.007% [5], and the mortality rate among hospitalizations is 8% [6]. In northern China, RSV infection is more common in winter and spring, while in southern China, it is more common in spring and summer, and RSV-infected patients are prone to repeated infection. It is reported that almost 100% of infants and young children will be infected with RSV at least once, and infected patients are prone to pulmonary diseases such as abnormal lung function and bronchial asthma after recovery [3, 7]. It is demonstrated that there is a strong association between asthma and severe RSV infection in early childhood. Besides, the elderly and immunocompromised individuals often develop severe lower respiratory tract disease after RSV infection.

Although the current RSV infection situation is very serious, due to the complex RSV infection mechanism, the different composition of the infected population, and the safety of its use, there is no safe and effective vaccine approved for marketing. The only drugs approved by the US FDA for the prevention and treatment of RSV infection are palivizumab and ribavirin. Palivizumab is a humanized monoclonal antibody, which is mainly used to prevent RSV infection in children with congenital heart disease and high risk of lung disease and premature, and cannot be used for post-respiratory syncytial virus infection treatment [8]. Moreover, the poor compliance and high cost of palivizumab also make it not the best choice for anti-RSV. Ribavirin, a broad-spectrum antiviral drug with the structure of guanosine analog, is currently the only small-molecule drug approved by the FDA for the treatment of RSV infection, but its efficacy and safety are still controversial [9]. Besides, there are some candidate RSV inhibitors in the clinical research stage, such as Lumicitabine (ALS-8176), Presatovir (GS-5806), PC-786, JNJ-678, and AK-0529. These RSV inhibitor candidates mainly target fusion protein F and RNA polymerase complex L protein, and some of which have completed clinical phase II, are expected to become new drugs for the treatment of RSV infection. However, the RSV fusion protein F inhibitor will appear cross-resistance, so the development of anti-RSV infection drugs still faces great challenges. The discovery of drugs with anti-RSV activity is imminent.

Studies [10, 11] have shown that many natural or chemically synthesized polysaccharides have inhibitory effects on various viruses. The polysaccharide from *Laminaria japonica* could exert its anti-RSV activity by up-regulating the production of IFN-*α* mediated by interferon regulatory factor 3 (IRF3) signaling, with an EC_50_ of 5.27 mg/mL and a therapeutic index (TI) value of 334 against RSV [10]. The polysaccharide component PSP-2B extracted from *Prunella vulgaris* showed excellent anti-HSV activity, and its IC_50_ for HSV-1 and HSV-2 was about 69 *μ*g/mL and 49 *μ*g/mL, respectively [11].

Jerusalem artichoke polysaccharide (JAP), a kind of inulin, is a chain polysaccharide composed of D-fructose linked by *β* (1–2) glycosidic bonds. Studies have shown that JAP has various effects including regulating gastrointestinal function, improving immunity, improving lipid metabolism, promoting mineral absorption, lowering blood lipids, and lowering blood sugar [12–17]. In this study, we evaluated the anti-RSV activity of JAP in *vitro* and *vivo* and further explored its potential mechanism of action.

## 2. Materials and Methods

### 2.1. Materials and Chemicals

Jerusalem artichoke was collected from Jingbian County, Shaanxi Province (China). Fructose standard was purchased from Shanghai Yuanye Biotechnology Co., Ltd. Other chemical regents are analytical grade and purchased from Sinopharm (Shanghai, China). RPMI 1640 (Biological Industries, Kibbutz Beit Haemek, Israel) containing 10% fetal bovine serum (Biological Industries, Kibbutz Beit Haemek, Israel) and 1% Penicillin-Streptomycin solution (Biosharp®, Anhui, China) was utilized as culture solution for Vero cells. Vero cells were cultured in CO_2_ incubator (HF90, Lishen Scientific Instrument Co., Ltd, Shanghai, China) at 37°C with a humidified atmosphere containing 5% CO_2_. Respiratory syncytial virus (RSV) and Vero cells were provided by collaborative innovation center of antiviral traditional Chinese medicine, Shandong University of traditional Chinese medicine. RSV was stored in medical cryogenic freezer (Haier, Shandong, China) at -80°C.

### 2.2. Extraction and Purification of JAP

The collected tubers of *Jerusalem artichoke* were washed and cut into homogeneous slices, and then extracted in hot water at 70-80°Cfor 1 h to obtain a crude extract containing JAP. The crude extract was sequentially purified by primary lime and then filtered to remove impurities such as peptides, proteins, and colloids. The prepurified extract was further refined, demineralized using cationic and anionic ion exchange resins, and decolorized using activated carbon. Finally, spray drying was performed to obtain JAP dry powder.

### 2.3. Determination of JAP Content

Total sugars were determined by the phenol-sulfuric acid method [18], and reducing sugars were determined by the 3,5-dinitrosalicylic acid (DNS) method [19]. The JAP content in the extract was the difference between the two.

### 2.4. Cytotoxicity Assay

To determine whether the inhibition of Vero cell proliferation was due to cytostatic or cytotoxic effects, the cytotoxicity of JAP *in vitro* was evaluated by MTT (3-(4,5-dimethylthiazol-2-yl)-2,5-diphenyltetr-azolium bromide) assay [20]. Briefly, JAP or ribavirin was added to Vero cells and placed in 96-well plates for 48 h, and then 20*μ*L MTT was added to each well and incubated for 4 hours to measure the absorbance of cells in each well at 490 nm by an Epoch 2T microplate reader. The maximum drug concentration at which cell viability was greater than 90% was recorded as TC_0_ (drug non-cytotoxic concentration).

(1) $$\begin{array}{c} \text{Cell survival rate}=\frac{\text{ODtest}-\text{ODblank control}}{\text{ODcell control}-\text{ODblank control}}, \end{array}$$

Cytopathic rate =1 − cell survival rate

OD_test_: The OD value of wells containing drug

OD_cell control_: The OD value of wells without drug

OD_blank control_: The OD value of wells containing cell culture medium only

### 2.5. Anti-RSV Activity of JAP *In Vitro*

JAP with the concentration of TC_0_ was added into a 96-well plate filled with monolayer Vero and then serially diluted 2-fold with RPMI-1640 containing 2% FBS to obtain 12 samples with different concentrations. 50 *μ*L of RSV with concentrations of 100 TCID50 were then injected into each well, and the blank control group (containing cell culture medium only), cell control group (containing cells), virus control group (containing cells and virus), and ribavirin group were set well. Each sample was assayed in triplicate. The 96-well plate was then placed in the incubator to observe the morphology and lesions of the cells. When 90% of the cells in the virus control group showed lesions, the culture was stopped. 20 *μ*L MTT dye solution and 50 *μ*L RPMI-1640 containing 2% FBS were added into each well after discarding the remaining culture medium in the 96-well plate and placed in a cell incubator for 4 h. 100 *μ*L of DMSO was then added for color development. Finally, measuring the optical density (OD) at 490 nm, the SI (selection index) value and IC_50_ (50% inhibitory concentration) were calculated by the Reed-Muench formula as follows:

(2) $$\begin{array}{c} pd=\frac{p1-50\%}{p1-p2},\\ {\text{CC}}_{50}\left(50\%\text{cytotoxicity concentration}\right)=\left[\text{Antilog }\left(\log A-pd\right)\right]\times Cm,\\ {\text{IC}}_{50}\left(50\%\text{inhibitory concentration}\right)=\left[\text{Antilog }\left(\log B-pd\right)\right]\times Cm,\\ \text{SI}\left(\text{selection index}\right)=\frac{\text{CC}50}{\text{IC}50}, \end{array}$$

*p*_1_: The cytopathic rate which is more than 50%

*p*_2_: The cytopathic rate which is less than 50%

*A*: The dilution ratio of the drug added to the well which has a cytopathic rate greater than 50%

*B*: The dilution ratio of the drug added to the well which has a cell survival rate greater than 50%

*C*_*m*_: Initial concentration of drug

### 2.6. Anti-RSV Activity of JAP *In Vivo*

#### 2.6.1. Grouping and Dosage Design

Sixty female Balb/c mice were randomly divided into six groups, including the blank control group, RSV model group, ribavirin group (0.1 g/kg), JAP low-dose group (0.5 g/kg), JAP medium-dose group (1.0 g/kg), and JAP high-dose group (2.0 g/kg). Except for the blank control group, mice in the other 5 groups received 50 *μ*L of RSV supernatant *via* the nasal drip. Two hours later, gavage was started, once a day. Each administration group was given 0.2 mL/20 g liquid medicine by gavage according to the body weight of mice, and the blank group and model group were given the same amount of 0.9% normal saline by gavage. After 5 days of continuous treatment, the administration was stopped. Weigh and record the weight of each mouse every day during the experiments. All the animal experiments were carried out in line with the regulations of the Animal Care and Use Committee at Shandong University of Traditional Chinese Medicine.

#### 2.6.2. Lung Index Inhibition Ratio and Histological Analysis

After 5 days of treatment with JAP, lungs from mice were subsequently dissected and weights to calculate lung index and lung index inhibition ratio. Paraffin sections of lung tissue were prepared and stained with hematoxylin and eosin (HE) stain. The samples were placed under an upright optical microscope (Nikon Eclipse E100, Japan) to obtain optical micrographs. Histopathological changes were described in results. Furthermore, the histological scoring system of lung tissue established in the previous literature (X. H. [21, 22]) was used to score the histopathological changes of lung tissue in this experiment. A final score (scale: 0-26) per mouse was obtained from assessing the quantity and quality of peribronchial and peribronchial inflammatory infiltrates, luminal exudates, perivascular infiltrates, and parenchymal pneumonia.

(3) $$\begin{array}{c} \text{Lung index}=\frac{\text{wet weight of lung}}{\text{body weight}}\times 100\%,\\ \text{Lung index inhibition ratio}=\frac{\text{Lung index of model group}-\text{Lung index of treatment group}}{\text{Lung index of model group}}\times 100\%. \end{array}$$

#### 2.6.3. Quantitative Real-Time Polymerase Chain Reaction (qRT-PCR)

The collected lung tissue was homogenized using a KZ-II homogenizer for RNA extraction to analyze the expression of three target genes RSV, TLR-3, and TLR-4 by qRT-PCR. Total RNA was isolated using Trizol reagent (Servicebio, Hubei, China). Reverse transcription reactions were performed using Servicebio® RT First Strand cDNA Synthesis Kit (Servicebio, Hubei, China) and Bio-Rad CFX. The reaction was incubated at 25°C for 5 minutes, followed by 30 minutes at 42°C, and finally at 85°C for 5 seconds. Analysis results were normalized to GAPDH. CT values were analyzed using the comparative CT (*ΔΔ*CT) method. Specific primers for qRT-PCR are listed in Table 1.

#### 2.6.4. Enzyme-Linked Immunosorbent Assay (ELISA)

The collected blood was centrifuged at 3 000 r/min for 15 min to obtain serum. TNF-*α*, TNF-*β*, and IL-4 concentrations were assessed with corresponding ELISA kits (Beijing 4A Biotech Co., Ltd, China) according to the manufacturer's instructions.

### 2.7. Statistical Analysis

Data are expressed as the means ± standard deviation (SD). One-way analysis of variance (ANOVA) for multiple groups was performed using GraphPad Prism 5.0 (GraphPad, La Jolla, CA, U.S.A.). A *P*-value of <0.05 was considered statistically significant.

## 3. Results

### 3.1. Extraction and Purification of JAP

After extraction and purification, the contents of total sugar and reducing sugar in the extract were measured to be 81.93% and 3.83%, respectively. After calculation, the JAP content in the extract was 78.10, and the yield was 11.73%. The JAP obtained in this assay has high purity with the doses of high related values in use.

### 3.2. Anti-RSV Activity of JAP *In Vitro*

The TC_0_ of JAP is about 10 mg/mL. According to the values of SI and IC_50_ index, JAP exhibited a similar inhibitory effect on RSV as ribavirin (Table 2).

### 3.3. Anti-RSV Activity of JAP *In Vivo*

#### 3.3.1. Body Weight Analysis

**Figure 1:**
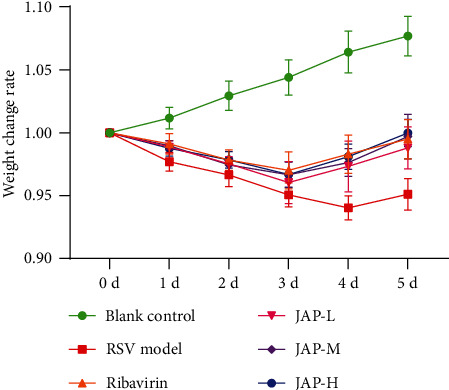
Effect of JAP on the body weight of RSV-infected mice (data were expressed as means ± SD, *n* =10).

Body weight change of mice (Figure 1) showed that 2 days after RSV infection, all groups (except the blank control group) experienced a significant weight loss. It was also observed that mice in the above groups were accompanied by symptoms such as decreased food intake, decreased activity, and a slight increase in body temperature, indicating that RSV successfully infected those mice.

The weight of mice in the blank group showed a steady increase. After drug treatment, the body weight of mice in ribavirin and each administration group began to recover on the fourth day, and there was no significant difference between the groups (*P* > 0.05). More importantly, the weight recovery rate of mice in the treatment group was significantly higher than that of the model group. The mice body weight changes suggested that JAP could significantly improve weight loss in mice induced by RSV infection.

#### 3.3.2. Lung Index Inhibition Ratio and Histological Analysis

**Figure 2:**
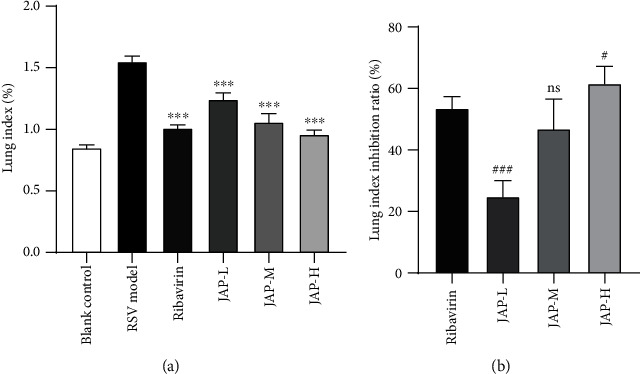
Effect of JAP on the lung index (a) and lung index inhibition ratio (b) of RSV-infected mice (data were expressed as means ± SD, *n* =10; ^∗∗∗^*P* value < 0.001 compared to RSV model group; ^###^*P* value < 0.001 compared to Ribavirin group; ^#^*P* value < 0.05).

As shown in Figure 2(a), the lung index of the RSV model group had significantly increased compared to the blank control group, indicating the mice were infected with RSV virus. Meanwhile, ribavirin and JAP treatment greatly reduced the lung index in mice (*P* < 0.001), suggesting both ribavirin and JAP attenuated RSV-induced lung injury. Moreover, the effect of JAP medium- and high-dose group was better than that in low-dose group, which was comparable to ribavirin from the results of the lung index inhibition rate (Figure 2(b)).

**Figure 3:**
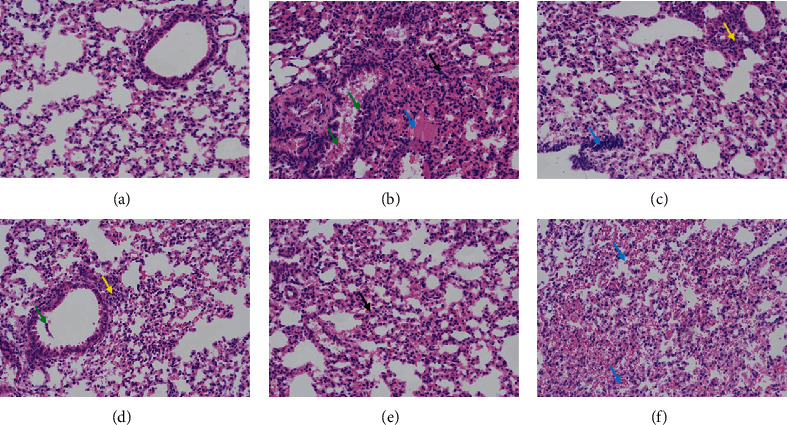
Histopathologic results of sections of the lung (200×). (a) Blank control group; (b) RSV model group; (c) Ribavirin group; (d) JAP low-dose group; (e) JAP middle-dose group; (f) JAP high-dose group.

**Figure 4:**
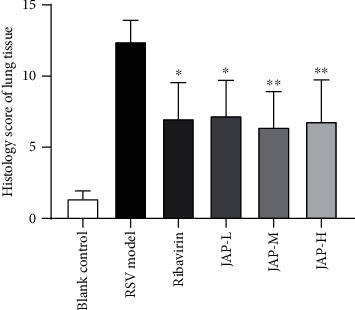
Histology score of lung tissue (data were expressed as means ± SD, *n* =5; ^∗∗^*P* value < 0.01 compared to RSV model group; ^∗^*P* value < 0.05).

Figure 3 shows the histology of lung tissue. Slight inflammatory cell infiltration was occasionally seen in the lung tissue without other obvious abnormalities in blank control group (Figure 3(a)). The lung tissue in RSV model group (Figure 3(b)) was to be found bronchiolar inflammatory exudates, epithelial cell pyknosis (green arrows), inflammatory cell infiltration, alveolar wall thickening (black arrows), and slight hemorrhage in alveolar spaces and eosinophilic coagulation (blue arrows). Mild pathological changes were also observed in lung tissues of both the ribavirin and JAP treatment groups. However, the histological scores of treatment groups shown in Figure 4 are significantly lower than that of the RSV model group, indicating that JAP treatment could effectively alleviate RSV-induced lung injury in mice.

#### 3.3.3. Effects of JAP on mRNA Expression of RSV, TLR3, and TLR4

**Figure 5:**
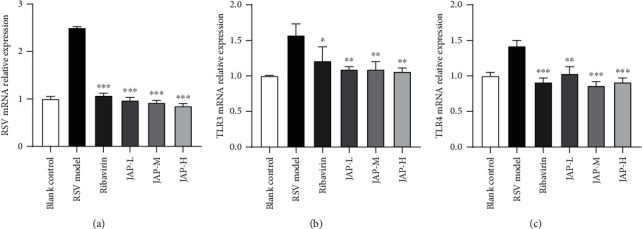
Relative RSV, TLR-3, and TLR-4 mRNA level (data were expressed as means ± SD, *n* =3; ^∗∗∗^*P* value < 0.001 compared to RSV model group; ^∗∗^*P* value < 0.01; ^∗^*P* value < 0.05).

To further confirm the anti-RSV activity of JAP in mice, the effect of JAP treatment on the RSV levels in lung tissue was evaluated by qRT-PCR. As shown in Figure 5(a), compared with the RSV model group, the mRNA levels of RSV in the lung tissue of the treatment groups were significantly reduced in a dose-dependent manner, confirming that JAP effectively inhibited the proliferation of RSV in the lung tissue and alleviated the RSV-induced lung damage.

Toll-like receptors, also known as pattern recognition receptors, play critical roles in many important biological functions, including the recognition of pathogens and activation of immune cascade signaling pathways. Recognition and binding of PAMPs by specific TLRs activate cellular signaling cascades through MyD88 (myeloid differentiation primary response 88)-dependent and MyD88-independent pathways, thereby inducing various cytokines, and thus induce inflammatory response [23–25]. To further explore the potential mechanism of JAP anti-RSV activity *in vivo*, the mRNA levels of TLR3 and TLR4 in lung tissue were also assessed by qRT-PCR. RSV infection significantly up-regulated the mRNA levels of TLR3 and TLR4 in lung tissue and stimulated pulmonary inflammatory response as shown in Figures 5(b) and 5(c). Both ribavirin and JAP treatment could decrease TLR3 and TLR4 mRNA levels, thus alleviating the inflammatory damage caused by RSV infection which was consistent with the results of histological analysis.

#### 3.3.4. Effects of JAP on the Levels of Cytokines in Serum

**Figure 6:**
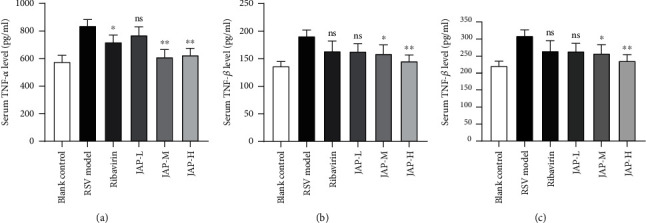
The content of TNF-*α*, TNF-*β*, and IL-4 in serum (data were expressed as means ± SD, *n* =4; ^∗∗^*P* value < 0.01 compared to RSV model group; ^∗^*P* value < 0.05).

The serum levels of pro-inflammatory factor TNF-*α*, TNF-*β*, and anti-inflammatory factor IL-4 were detected by ELISA to further explore the anti-RSV mechanism of JAP. As shown in Figure 6, the levels of TNF-*α* and TNF-*β* were significantly decreased in the middle- and high-dose groups after 5 days of JAP treatment (*P* < 0.05). However, the content of IL-4 in serum was only significantly increased in the high-dose JAP group (*P* < 0.05), which might be related to the dose. It is concluded that JAP might regulate the level of cytokines by inhibiting the levels of TLR3 and TLR4, thereby intervening in the inflammatory response process of infected mice.

## 4. Discussion

RSV is the main cause of lower respiratory tract infection in infants, the elderly, and the immunocompromised. Furthermore, severe respiratory disease induced by RSV infection is associated with an increased risk of long-term wheezing and later asthma [26]. Although RSV vaccines have been developed for more than 50 years, there are still no vaccines approved for marketing. Currently, there are no vaccines and effective therapeutic drugs for the prevention or treatment of RSV infection in clinical practice.

JAP is an inulin-type polysaccharide extracted from *Jerusalem artichoke*, which has the functions of regulating gastrointestinal function, improving immunity, improving lipid metabolism, promoting mineral absorption, lowering blood lipids, and lowering blood sugar [12–17]. Importantly, many studies have confirmed that polysaccharides also have antiviral activity [10, 11, 27–29]. The effect of JAP on RSV has not been previously reported. Here, we isolated JAP from *Jerusalem artichoke* and evaluated its antiviral activity against RSV *in vitro*. It was demonstrated that JAP had an anti-RSV SI of 68.27, showing an anti-RSV level comparable to that of ribavirin. Additionally, we also assessed the anti-RSV effect of JAP *in vivo*. The results suggested that JAP not only significantly improved the weight loss of RSV-infected mice in a dose-dependent manner (Figure 1) but also effectively attenuated RSV-induced lung injury in mice (Figures 2 and 3). More importantly, JAP significantly reduced the level of RSV mRNA, indicating that JAP inhibited the proliferation of RSV in lung tissue.

TLRs are known as the first line of defense against invading pathogens by recognizing and triggering a suitable response against pathogenic attacks, which play an integral role in inflammation, immune cell regulation, survival, and proliferation [24]. A total of 11 TLRs have been found in humans so far. Among them, TLR4 mainly recognizes the LPS component of Gram-negative bacteria and the fusion protein of RSV, and TLR3 mainly recognizes dsRNA produced during virus replication. Studies have shown that both TLR4 and TLR3 pathways are involved in RSV-induced airway epithelial cellular inflammatory response [30, 31]. Overexpression of inflammatory cytokines such as TNF-*α* is an important factor in causing acute inflammatory damage to the lung. IL-4 is recognized to have both anti-inflammatory and immunomodulatory effects. Here, we found that the JAP treatment group could significantly reduce the mRNA levels of TLR-3 and TLR-4, down-regulate the expressions of pro-inflammatory factors TNF-*α* and TNF-*β*, and up-regulate the level of anti-inflammatory factor IL-4. The above results suggested that JAP may affect the downstream inflammatory factor levels by inhibiting the expression of TLR3 and TLR4, thereby intervening in the inflammatory response process of infected mice.

JAP, a type of inulin, is a recognized natural prebiotic, which is fermented in the gut to produce a large amount of short-chain fatty acids and significantly increase the number of bifidobacteria, thereby improving the intestinal flora [32, 33]. Furthermore, studies had shown that changes in the composition of gut microbiota and changes in the concentration of short-chain fatty acids in the gut were associated with RSV susceptibility [34, 35]. It is reported that JAP could also enhance intestinal mucosal immune function, and its underlying mechanism was related to the changes in intestinal flora caused by inulin fermentation [16, 36]. Based on the above studies, we also speculate that the intervention effect of JAP on RSV-induced pneumonia in mice might be related to its effects on intestinal mucosal immunity and intestinal flora. Therefore, the follow-up mechanism research will focus on intestinal mucosal immunity and changes in the intestinal flora. In addition to an in-depth study of its anti-RSV mechanism, we will also systematically evaluate the safety of JAP to lay the foundation for this study to enter human trials.

In conclusion, we preliminarily confirmed the anti-RSV effect of JAP *in vitro* and *in vivo* and found that its mechanism involved inhibiting TLR3/4 mRNA levels, down-regulating pro-inflammatory factors TNF-*α* and TNF-*β* levels, and up-regulating anti-inflammatory factor IL-4 levels. These data suggest the potential use of JAP as antiviral agent against RSV. However, further studies are needed to elucidate their mechanisms, which remain unclear.

## Figures and Tables

**Table 1:** Specific primers used for qRT-PCR.

Target gene	Primer sequences	
	Sense	Anti-sense
RSV	5′-TGGGACACTCTTAATTAATCAT-3′	5′-TGATTCCAAGCTGAGGAT-3′
TLR3	5′-ACCTTTCCGCCCTCTTCGTAAC-3′	5′-TTCTCAAGACCCTCCAGCAAGTC-3′
TLR4	5′-CTGCAATGGATCAAGGACCA-3′	5′-TTATCTGGTGTTGCACATTCC-3′
GAPDH	5′-AGGTCGGTGTGAACGGATTTG-3′	5′-GGGGTCGTTGATGGCAACA-3′

**Table 2:** Inhibition of JAP on RSV.

Drug	CC_50_ (*μ*g·mL^−1^)	IC_50_ (*μ*g·mL^−1^)	SI
Ribavirin	2301.03 ± 10.24	30.19 ± 0.23	76.21 ± 0.37
JAP	1989.35 ± 33.11	29.15 ± 0.44	68.27 ± 1.77

## Data Availability

The data used to support the findings of this study are available from the corresponding author upon reasonable request.
