# Correction to “Antiviral Activity Against Respiratory Syncytial Virus of Polysaccharide From Jerusalem Artichoke (*Helianthus tuberosus* L.)”

**DOI:** 10.1155/bmri/9849657

**Published:** 2025-10-21

X. Wan, Z. Liu, Y. Wang, et al., “Antiviral Activity Against Respiratory Syncytial Virus of Polysaccharide From Jerusalem Artichoke (*Helianthus tuberosus* L.),” *BioMed Research International* 2022 (2022): 1809879, https://doi.org/10.1155/2022/1809879

In the article, during the production process, an error was introduced in Figure 6c, whereby the *y* *-*axis label should read “Serum IL‐4 level (pg/mL).” The correct Figure 6 is shown below:

Figure 1The content of TNF‐*α*, TNF‐*β*, and IL‐4 in serum (data were expressed as means ± SD, *n* = 4;  ^∗∗^ *p* value < 0.01 compared to the RSV model group;  ^∗^ *p* value < 0.05).

**Figure:**
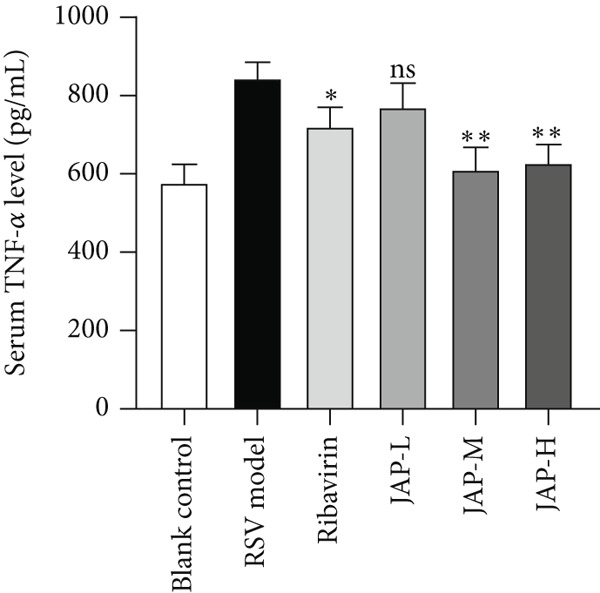
(a)

**Figure:**
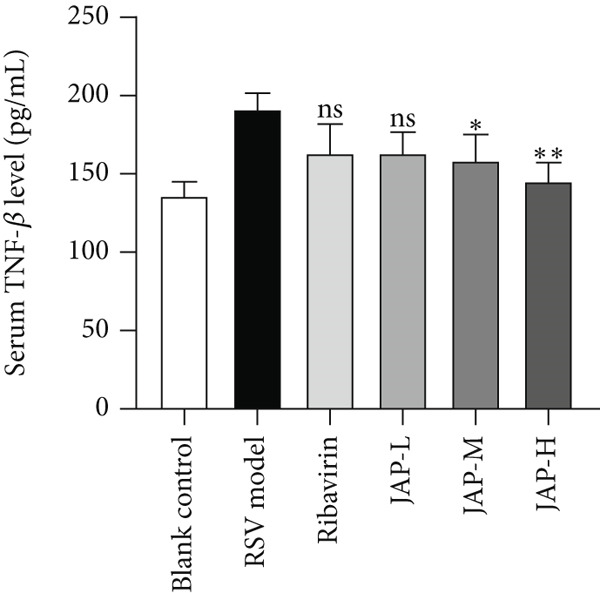
(b)

**Figure:**
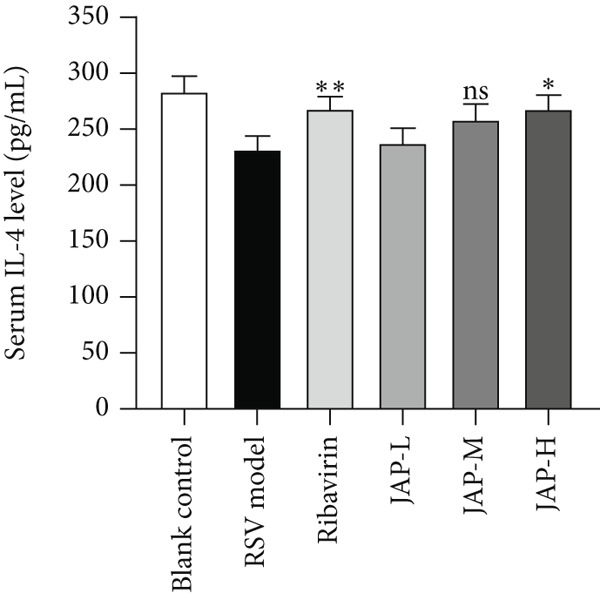
(c)

We apologize for this error.

